# Efficiency of the anaerobic digestion of amine wastes

**DOI:** 10.1007/s10529-013-1296-1

**Published:** 2013-08-04

**Authors:** Shuai Wang, Jon Hovland, Rune Bakke

**Affiliations:** 1 Department of Process, Energy, and Environmental Technology, Telemark University College, Kjølnes Ring 56, Postboks 203, 3910 Porsgrunn, Norway; 2 Tel-Tek, Kjølnes Ring 30, 3918 Porsgrunn, Norway

**Keywords:** Ammonia, Anaerobic digestion, CO_2_ capture, Ethanolamine, Monoethanolamine, Principle component analysis

## Abstract

Laboratory-scale anaerobic degradation of monoethanolamine waste (MEAw) with co-substrate organics was conducted at room temperature and organic loading rates from 0.19 to 5.03 kg COD/m^3^ day for 486 days in a hybrid digester. 90 % feed COD conversion to methane was obtained at the lower loads and only 45 % at the highest MEA waste/COD ratio (MEAwr) of 0.62 due to inhibition of methanogenesis. Inhibition at comparable loads decreased with time, implying that the culture adapted to the challenging feed. Methane yield was negatively correlated to MEAwr applied and inhibition avoided at MEAwr <0.5. Acetate accumulation implies inhibition of acetoclastic methanogenesis that can be caused by ammonia, a product of MEAw degradation. Moderate total ammonia nitrogen and free ammonia nitrogen accumulation, maximum 2.2 g N/l and 90 mg N/l, respectively suggests, however, that other components of MEAw, and/or degradation products of such, also inhibit methanogenesis, disturbing the digester performance.

## Introduction

There has been increasing focus on CO_2_ capture (CC) as a measure to mitigate greenhouse gas emissions. In CC, alkaline amine solvents used for absorption of CO_2_ [e.g. monoethanolamine (MEA)] is considered to be the most mature technology (IEA 2012). MEA and other alkanolamines are also commonly used for the absorption of acidic gases (CO_2_, H_2_S etc.) from natural gas and in refineries. The solvents are repeatedly used by regeneration through distillation in the capture process (Barchas and Davis 1992). Over time, the amine solvents degrade due to oxidation, thermal degradation, carbamation and reaction with SO_x_, NO_x_ and dust in the flue gas as well as by other means (Goff and Rochelle 2004; Davis and Rochelle 2009). A concentrated solution of reclaimer waste accumulates at the bottom of the reclaimer facility after distillation. This concentrated solution is classified as hazardous waste and must be stored and disposed of accordingly.

Biological MEA waste (MEAw) treatment has been suggested and investigated (Hauser et al. 2013; Botheju et al. 2011; Wang et al. 2013). Anaerobic digestion (AD) is a means to both break-down wastes and recover energy from it as methane. AD is a synergistic biological process that involves various types of organisms. AD of MEAw is challenging due to its relatively high N:C ratio and high content of complex chemicals. Inhibition and low chemical oxygen demand (COD) removal efficiency was observed at high loading during co-digestion of MEAw with easily degradable organics (Wang et al. 2013). AD is, though, vulnerable to toxic effects from ammonia and feed organics (Chen et al. 2008). Ammonia is a degradation product of MEA that can be inhibitory to the AD process (Hansen et al. 1998). Additionally, chemicals from MEAw may also stress the organisms.

The aim of this study was to examine the anaerobic degradation of MEAw to determine the limitations of waste loading. The study is a continuation of a previously published experiment (Wang et al. 2013) running the reactor for more than a year to investigate how waste degradation limitations change as the culture adapts to the feed. The AD capacity was tested by measuring the methane yield, COD removal efficiency and ammonia and volatile fatty acid (VFA) accumulations. Principle component analysis (PCA) was applied to investigate the relative significance of possible inhibitory compounds.

## Materials and methods

### Feed and nutrient

Reclaimer monoethanolamine MEA waste with combined easily-degradable organics was applied as feed substrate (Table 1). The MEAw investigated was collected from a full-scale MEA-based, CO_2_ capture facility at a coal-fired power plant. The waste contained complex components that were not well identified. The measured COD of the waste varied between 450 and 900 mg COD/g waste, the N content was ~7–14 % (w/w) and the MEA accounted from 18 to 30 % (w/w). Detailed information about similar wastes can be found in Strazisar et al. (2003) and Thitakamol et al. (2007).

**Table 1:** Compositions of the feed substrate added during the experimental period

Component	Concentration (g/l)	COD (g COD/l)	Nitrogen concentration (g/l)
Starch (glucose)^a^	1.5 (1.7)	1.8	0
Yeast extract	3.6	3.3	0.4^b^
Peptone	3	4.5	0.4^c^
MEA waste	4–25	1.7–15.6	0.6–3.5^d^
Total	12.1–33.1	11.3–25.2	1.4–4.3

^a^Starch was replaced by glucose at 250th day

^b^Product reference shows a nitrogen concentration of 10.5 % (w/w) in this yeast extract

^c^Product reference shows a nitrogen concentration of 12–13 % (w/w) in this peptone. 12.5 % (w/w) was used in this calculation

^d^An approximate N fraction of 14 % (w/w) was measured and used here

Starch, replaced by glucose after 250 days (due to the detection of starch accumulation on the inner wall of the feeding pipe, to avoid flow disturbances and inconsistent mass balances that it could cause), peptone and yeast extract mixture was applied as the co-substrate feed. The co-substrates were used to provide necessary nutrients, minerals and various easily degradable organics to maintain biomass that can tolerate exposure to toxic and inhibitory chemicals from the MEAw. Preliminary but unpublished tests show that methanogenesis cannot be maintained on MEAw alone as feed substrate. Constant concentrations of first starch, then glucose, peptone and yeast extract were used in the whole test period (Table 1). KH_2_PO_4_ (0.15 g/l) and K_2_HPO_4_ (0.15 g/l) were also added to the feed as buffers.

Feed solutions were prepared by mixing the MEAw and co-substrate in deionized water and stored at 4 °C. The pH of the feed mixture was around 10–11 (varying depending on the MEAw content).

10 ml buffer (102 g KH_2_PO_4_/l; 131 g K_2_HPO_4_/l) and mineral solutions (Table 2) were added to the system at the start-up of the digester to stabilize pH and provide the necessary minerals in the pre-adaptation period. No external buffer and minerals were supplied after the initial addition.

**Table 2:** Mineral solution composition used at the startup of the digester

Chemical	Value (mg/l)
MnSO_4_·H_2_O	40
FeSO_4_·7H_2_O	2,800
CuSO_4_·5H_2_O	60
NiCl_2_·6H_2_O	92
ZnSO_4_·7H_2_O	90
CoCl_2_·6H_2_O	50
H_3_BO_3_	50
(NH_4_)_6_Mo_7_O_24_	50
AlCl_3_	50
Na_2_SeO_3_·5H_2_O	50
EDTA	100

### Biomass

A mixture of inocula from various sources were added in the reactor at the commencement of the test. 200 ml of settled fresh granular sludge, with relatively equal size (~2 mm) from a pulp and paper industrial wastewater treatment Upflow anaerobic sludge blanket reactor (UASB) in Norway, was applied as the main inoculum. Some polluted river bed sludge (Lilleelva river in Porsgrunn, Norway) and biomass from other laboratory experimental tests (aerobic and anaerobic reactors treating domestic wastewater) were also added in the reactor to give higher biomass diversity. No taxonomical classification was carried out. No extra biomass was added after the experiment startup.

### Experimental set-up

**Fig. 1:**
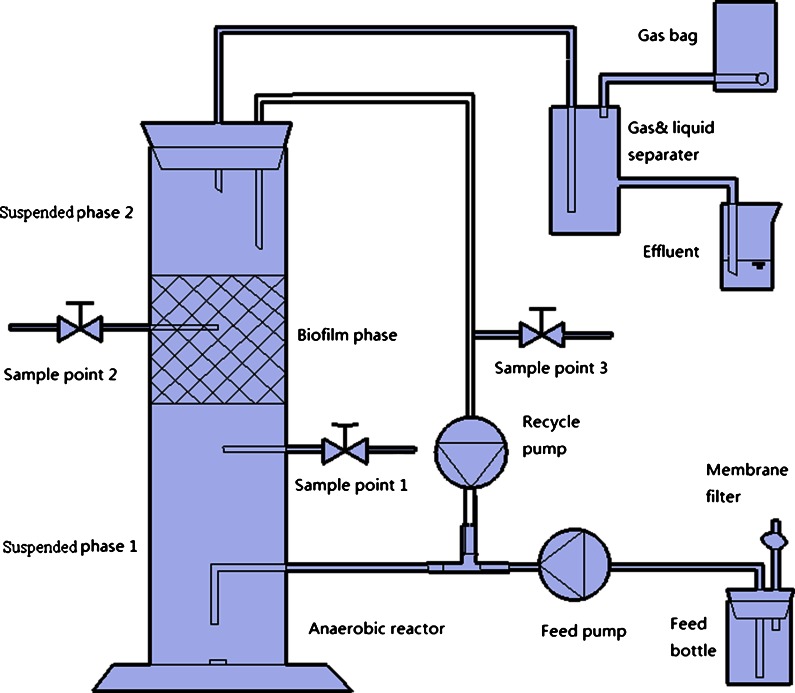
Schematic of the used anaerobic hybrid reactor system (Wang et al. 2013)

The applied anaerobic reactor set-up is shown in Fig. 1. The reactor was made from a transparent acrylic tube, which was divided into two suspended fluidized bed phases by a fixed biofilm phase, making it a hybrid reactor combining attached and suspended biomass. The total working volume was 1.25 l. Magnetic stirring was employed in the bottom suspended phase (0.8 l) to avoid sludge sedimentation and “dead zones” at the reactor bottom. Feed was pumped intermittently into this phase using a peristaltic pump. The 0.15 l biofilm phase contained a porous rock material (Light Expanded Clay Aggregates, “Leca” from Weber, Saint-Gobain) as the biofilm substratum contained within a plastic net. The upper suspended phase (0.3 l) was intended as a second sludge bed reaction zone and sedimentation zone to retain sludge in the reactor. The reactor design was intended to achieve long solid retention times compared to hydraulic retention time to enable efficient cultivation of MEAw consuming biomass and allow relatively high OLR. A recycle rate of 25 ml/min was maintained by a peristaltic pump to fluidize the sludge.

### Experimental management

The experiment was performed at 22 ± 2 °C. A pre-adaptation period of three months was applied first to adapt the inoculum to the feed substrate with MEAw. Then, 486 days of experimental data were collected and analyzed. The total feed COD concentrations applied varied in a range of 10–25 g/l, of which MEAw was 1.7–15 g COD/l. The co-substrate had a constant concentration of ~9.6 g COD/l (Table 1).

**Fig. 2:**
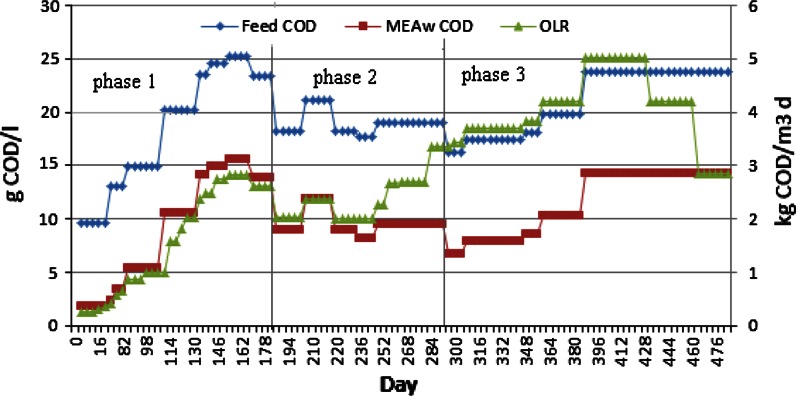
Feed and MEA waste COD concentration and organic loading rate (OLR) applied in the experiment test

In the pre-adaptation, MEAw concentration in the feed and OLR were kept low and increased carefully (upto ~4 g MEAw/l) to avoid biomass loss due to toxic and inhibitory effects. This adaptation period ended when continuous and relatively stable biogas generation was observed. After pre-adaptation, the experimental test was continuously operated with three distinct phases of different feeding strategies (Fig. 2). In phase 1 (0–181 days), MEAwr in feed COD was increased from 0.18 to a maximum of 0.62, the corresponding OLR applied was from 0.25 to 2.82 kg COD/m^3^ day. This period was designed to get an overview of the digestibility of MEAw substrate and system capacity and data analysis on this period is published by Wang et al. (2013). Process stability was investigated in phase 2, where MEAwr was kept at around 0.5 (182–281 days) and the OLR was in the range of 2–2.62 kg COD/m^3^ day. High loads were tested in phase 3 (282–486 days), where MEAwr was elevated from 0.4 to 0.6 and OLR from 2.66 to a maximum of 5.03 kg COD/m^3^ day. OLR was reduced to 2.86 kg COD/m^3^ day while maintaining MEAwr at 0.6 at the end of phase 3. The feed COD variations were imposed by changing MEAw concentrations while the co-substrate COD concentrations remained constant (Table 1). The applied loads were varied by modifying either the MEAwr or increasing the feed loading (flow) rate.

The generated biogas was collected continuously in a gas bag (Cali-5-Bond) during the experiment (Fig. 1). The biogas volume and its composition were measured every 2 days. Liquid samples were collected from sample point 3 every 2 days (Fig. 1) for the measurements of pH, VFA, ammonia and soluble COD (sCOD) concentrations. The pH was analyzed for each sample, while the analysis of VFA, ammonia and COD were conducted for every other sample due to the relatively small amount of samples collected.

### Analytical methods

The analytical methods used for the measurements of VFAs, biogas composition, COD, MEA and ammonia concentrations and the calculation of total and free ammonia nitrogen concentration can be referred to Wang et al. (2013).

Principal component analysis, PCA, is a statistical technique to identify patterns in data, and express the data in a way to highlight similarities and differences (Abdi and Williams 2010). Correlation circle generated from PCA analysis was used to interpret the correlation between different variables used. Commercial Excel add-in software XLSTAT was used for the PCA analysis.

## Results and discussion

The 486 days of anaerobic digester performance data were recorded and analyzed by assessing the COD and MEA waste removal efficiency and methane yield, showing that the extent of methane generation from waste degradation varied significantly. The influences of operational conditions and inhibition factors on anaerobic MEAw degradation are examined. Data from the first part of this experiment, published elsewhere (Wang et al. (2013)), are repeated here to more clearly show the digestion efficiency development during a long time span and compare results from a wide range of load conditions tested.

### COD removal

**Fig. 3:**
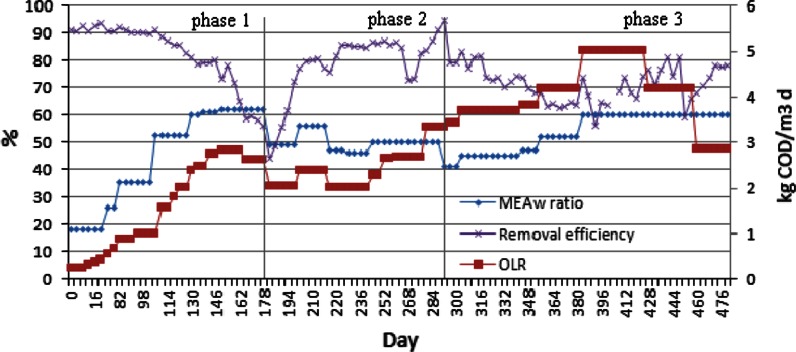
COD removal efficiency (%) under different feed OLR and MEA waste ratios in feed

The COD removal efficiency based on the influent and effluent soluble COD concentration measurements are given in Fig. 3. The COD removal efficiency was generally above 90 % before day 108. Afterwards, it gradually decreased to 45 % when the applied MEAwr increased above 0.5 to a maximum of 0.6 with OLR in the range from 1.5 to 2.8 kg COD/m^3^ day (109–181 days). In light of the apparent impending system failure, MEAwr was reduced to around 0.5 while the OLR was maintained between 2 and 3 kg COD/m^3^ day in phase 2 (182–282 days). During this period, COD removal efficiency recovered to about 80 % in one month and then remained relatively constant at MEAwr of 0.5 and an OLR of about 2.5 kg COD/m^3^ day.

In phase 3, the COD removal efficiency reached up to 90 % again from 283 to 292 days, at a MEAwr of 0.5 and OLR of 3.43 kg COD/m^3^ day. The relatively high efficiency at a much higher OLR than that applied in phase 1 indicated that high OLR in the later part of phase 1 (2.82 kg COD/m^3^ day) was not the reason of the decreased removal efficiency. A stepwise increase of OLR to 5.03 kg COD/m^3^ day by increasing feed MEAwr from 0.4 to 0.6 decreased the removal efficiency again to about 65 % (293–428 days). This decline reveals that the increase of MEAwr does negatively affect the removal efficiency. High MEAwr is evidently more challenging for the involved organisms than high MEAw loading rate. The system has high capacity in terms of organic loading.

A comparison of removal efficiency at a similar feed MEAwr to that applied in phase 1 around 160 days and after 400 days shows that the removal efficiency was higher in the latter case even at higher loading rates. It indicates that the organisms continued to adapt to the complex feed substrate and inhibitory factors that this feed may have presented or inhibitory products of MEAw degradation (e.g. NH_3_) through the whole test.

### Mass balance

**Fig. 4:**
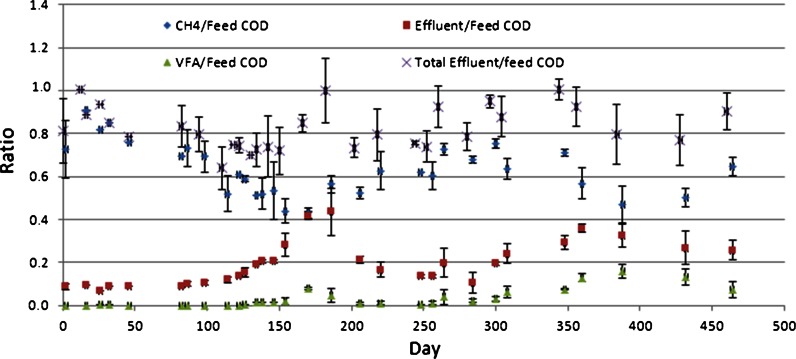
Ratios of methane, VFA and effluent soluble COD to the total feed COD. *Error bars* represent one standard deviation

Mass balance for the main COD constituents, calculated at each sampling time based on the measured average methane generation, effluent VFA and effluent sCOD concentrations are shown in Fig. 4. Data, normalized to total feed COD, shows that 80–90 % of the feed COD was recovered as methane and sCOD. A varying fraction of the sCOD was VFA, reaching a maximum of ~50 % during the highest load in phase 3. The COD not accounted for in Fig. 4 (when total effluent/feed COD <1) can be produced biomass accumulating in the reactor and/or leaving as particulate COD. Observed sedimentation of some feed starch on the wall of the feeding pipe may have contributed to loss of feed COD and mass balance errors. Glucose was used instead of starch after 250 days in the feed solution to avoid this potential error. Afterwards, methane COD and effluent sCOD added up to approx. 0.9 times feed COD, showing that 10 % of the feed COD was converted to biomass and some was probably lost as recalcitrant chemicals absorbed to particles/biomass.

**Fig. 5:**
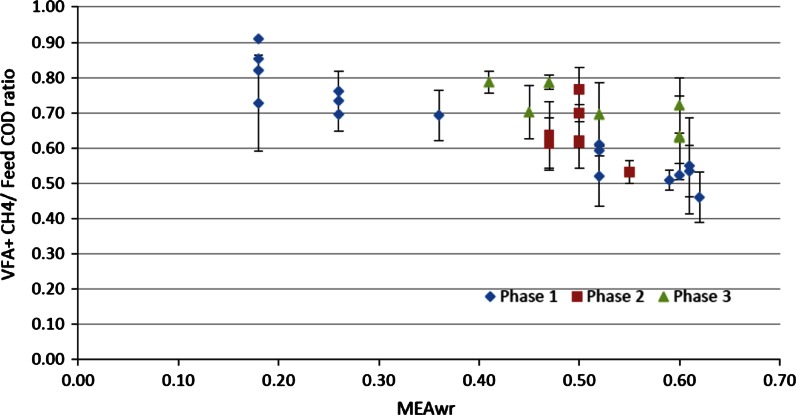
Combined VFA and methane COD to the feed COD ratio under different applied MEA waste COD ratios (MEAwr) in feed in the tested three phases; *Error bars* represent one standard deviation

Figure 5 shows the combined methane and VFA COD to feed COD ratio with respect to applied MEAwr in feed to show how much of the feed was hydrolyzed and acidified (termed degradation ratio). Data from phase 1 shows that the degradation ratio decreased by a linear trend from about 0.8–0.5 at the maximum MEAwr applied. Only about 50 % of the feed COD was broken down at the highest MEAwr. The degradation ratio increased during the long adaptation period in phase 2 while maintaining relatively high MEAwr. After that the degradation efficiency during phase 3 was consistently higher than in phase 1 in the MEAwr range (0.4–0.6) tested. This clearly demonstrates the biomass adaptation to feed MEAw discussed above. The fraction of undetected substances in the feed that were reduced and degraded to methane and VFA still increased after hundreds of days of reactor operation.

### pH and ammonia

**Fig. 6:**
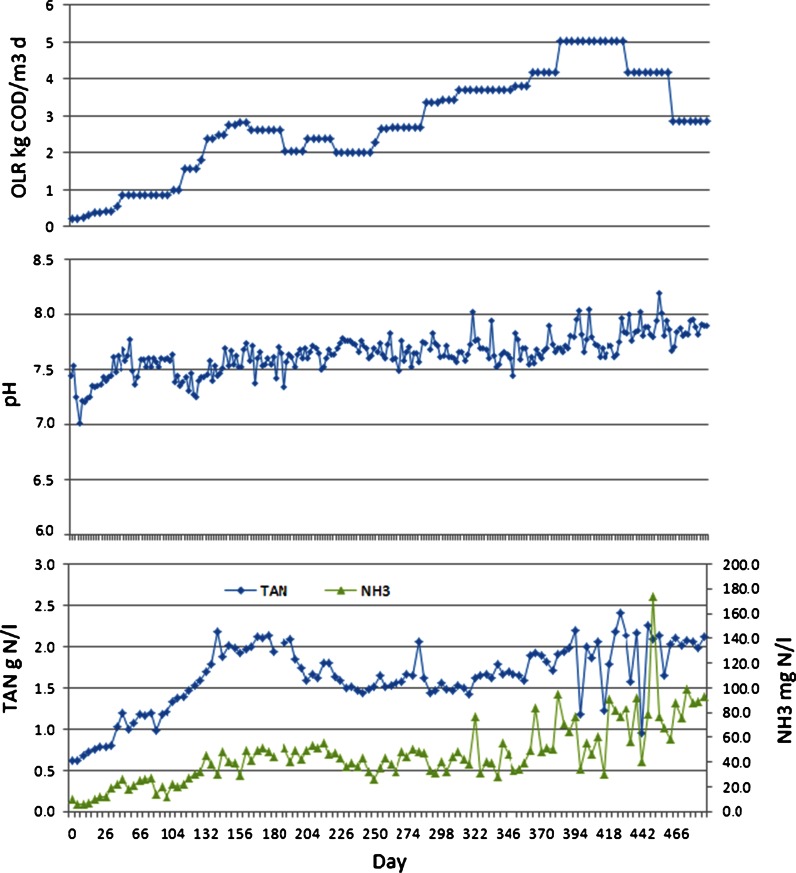
Reactor effluent pH and total and free ammonia nitrogen (TAN and FAN) concentrations under varying feed organic loading rate (OLR) during the test period

**Fig. 7:**
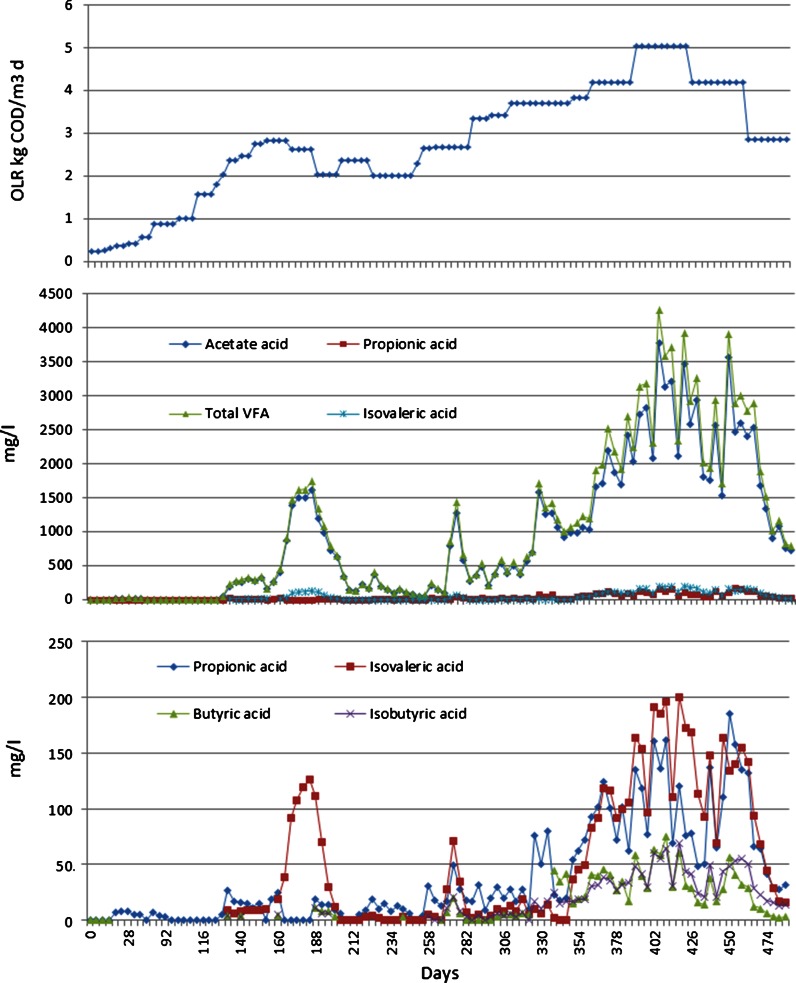
Volatile fatty acid (VFA) accumulations under varying feed organic loading rate (OLR)

The MEAw used had high alkalinity (16 g CaCO_3_/l equivalent, for a 50 g MEAw/l solution in distilled water). The feed solution pH varied depending on the MEAw concentration and reached 10.5 when 25 g MEAw/l was applied. The digester effluent pH was measured in the range of 7.0–8.0 in the whole test period without applying pH adjustment in the AD. Even at the highest VFA accumulation (402–420 days, Fig. 7) the pH was well above 7, attributed to the high alkalinity buffer capacity of the feed solution as well as the accumulated ammonia concentration (Fig. 6). The effluent VFA concentrations varied from 0 to a maximum of 4 g/l and total and free ammonia nitrogen (TAN and FAN) reached maximums of 2.2 g/l and 90 mg/l, respectively (Figs. 6, 7).

Figure 6 shows the ammonia concentration variations in the digester for the whole test period. The TAN concentration reached a maximum of about 2.2 g N/l at around 181 days and the highest FAN was 50 mg N/l in phase 1. TAN and FAN concentrations decreased simultaneously as OLR and MEAwr reduced and then stayed relatively constant in phase 2. Ammonia concentration increased again when feed MEAwr increased in phase 3. TAN and FAN varied more in phase 3 and were 2.2 g N/l and 90 mg N/l, respectively, at the end of the test.

### Inhibition

Acetate was more than 90 % of the VFA accumulated in the digester with minor accumulations of several other fatty acids. Isovaleric, propionic, butyric and isobutyric acid were detected when acetate concentrations were high (Fig. 7).

VFA accumulated when the methane yield decreased, implying that inhibition of VFA consuming organisms is a reasonable explanation of the diminished methane yield during periods of high MEAwr. Acetoclastic methanogenesis is evidently especially sensitive to the factors causing the inhibition since most of the accumulated VFA was acetate (Fig. 7). High concentrations of free ammonia can also cause such inhibitions, but the levels observed here were low compared to those reported to cause inhibition (e.g. observed inhibition coefficient K_i_ (50 %) is 30–90 mg N/l) (Gallert et al. 1998; Batstone et al. 2002). High tolerance of 800 mg N/l was observed (Calli et al. 2005). It therefore, seems likely that some other constituents of MEAw and/or degradation products thereof contribute significantly to the observed inhibition. Complex and recalcitrant chemicals from MEAw may inhibit the anaerobic degradation and cause VFA accumulation. Anaerobic microbial consortia can adapt to high ammonia by establishing an alternative pathway (Schnürer et al. 1994). The observation of less VFA accumulation at similar feed MEAwr and OLR (0.6 and 2.82 kg COD/m^3^ day) at the end of phase 3 compared to phase 1 (Fig. 7) suggests that such an adaptation may have occurred. This is supported by the observation that TAN was at a similar level in those two feed conditions while FAN concentrations were at a higher (90 mg/l comparing to 50 mg/l) level due to higher pH in phase 3 (Fig. 6). If FAN was the main inhibitory factor in phase 1, it indicates that the biomass consortium gradually acclimated to the inhibitory ammonia, possibly by establishing the alternative pathway described by Schnürer et al. (1994).

**Fig. 8:**
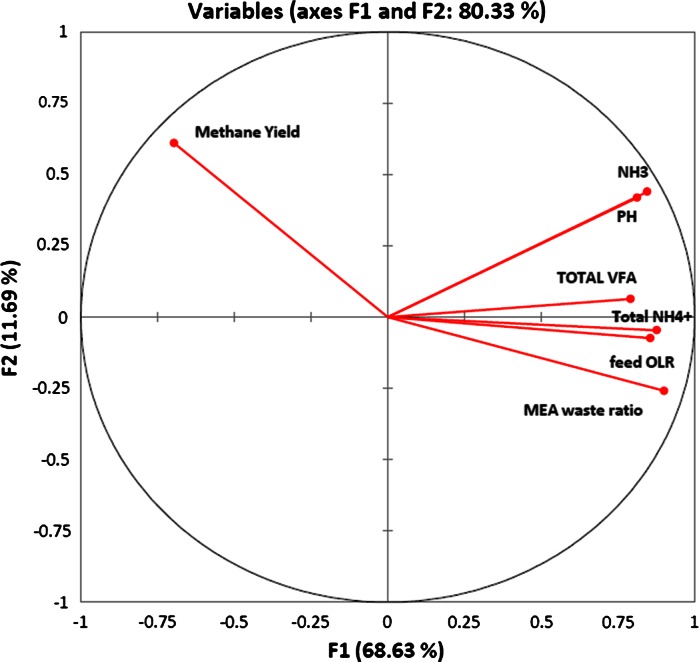
Correlation circle (below on axes F1 and F2) shows a projection of the initial variables in the factors space, indicating the correlations between different variables. When two variables are far from the origin, if they are close to each other, they are significantly positively correlated; when they are opposite from each other, they are negatively correlated; when they are orthogonal to each other, they are not significantly correlated

Figure 8 shows the correlation circle from PCA, where a projection of the initial variables (methane yield, pH, NH_3_, total VFA, feed OLR, total NH_4_^+^ and MEAwr) are shown in the factors space. The correlation between variables can be interpreted through the angles of variable projections. When two variables are far from the origin and they are close to each other, such as pH and NH_3_, they are significantly positively correlated (r close to 1). Methane yield is almost orthogonal to pH and NH_3_, implying that they are not significantly correlated (r close to 0). MEAwr is on the opposite side of the center from methane yield, implying that they are significantly negatively correlated (r close to –1). OLR and total ammonia which varied with the MEAwr applied are also quite on the opposite side of Methane yield, implying that these two variables may also strongly affect the methane yield. Total ammonia concentration and accumulated VFA may also cause stress in the methanogenic pathways and contribute to variations of the methane yield (Lü et al. 2013).

MEAwr played an important role in providing feed nitrogenous organics and thereby contributed to the ammonia accumulation when MEAw was degraded. PCA shows that MEAwr in feed is the most important impact negative factor to the methane yield, more so than FAN and TAN (Fig. 8). This supports the claim that some other constituents of MEAw and/or degradation products from such are the cause of, or contribute significantly, to the observed inhibition.

## Conclusion

Anaerobic degradation of MEAw with easily degradable co-digestible substrates was conducted in a hybrid anaerobic digester for 486 days at room temperature. The mixed feed substrate degradation ratio reached a maximum of 93 % when no inhibition effects were observed at low MEAwr (<0.5) and OLR <3 kg COD/m^3^ day. Principal component analysis showed a strong negative correlation between MEAwr and methane yield in the load range tested, MEAwr >0.5 reduced methane yield. VFA, mainly as acetate, accumulated with high MEAwr causing inhibitory conditions implying that acetoclastic methanogenesis was the step that was mainly inhibited. Complex and recalcitrant chemicals in MEAw and products of degradation, including ammonia, may be the cause of the inhibited AD performance. Significantly less inhibition was observed after a year of operation compared to the first phase of the experiment implying some microbial adaptation to the inhibiting factors. High MEA waste removal efficiency by AD can thus be achieved by cultures adapted to such feed in an appropriate co-substrate mixture. Relatively stable system performance can be obtained given moderate load changes.
